# IRAK4 Deficiency Presenting with Anti-NMDAR Encephalitis and HHV6 Reactivation

**DOI:** 10.1007/s10875-020-00885-5

**Published:** 2020-10-20

**Authors:** Shiho Nishimura, Yoshiyuki Kobayashi, Hidenori Ohnishi, Kunihiko Moriya, Miyuki Tsumura, Sonoko Sakata, Yoko Mizoguchi, Hidetoshi Takada, Zenichiro Kato, Vanessa Sancho-Shimizu, Capucine Picard, Sarosh R. Irani, Osamu Ohara, Jean-Laurent Casanova, Anne Puel, Nobutsune Ishikawa, Satoshi Okada, Masao Kobayashi

**Affiliations:** 1 Department of Pediatrics, Hiroshima University Graduate School of Biomedical and Health Science, 1-2-3 Kasumi, Minami-Ku, Hiroshima-Shi, Hiroshima, 734-8551 Japan; 2 Department of Pediatrics, Graduate school of Medicine, Gifu University, Gifu, Japan; 3 Laboratory of Human Genetics of Infectious Diseases, Necker Branch, INSERM UMR, 1163 Paris, France; 4 Present Address: Department of Pediatrics, Tohoku University Graduate School of Medicine, Sendai, Japan; 5 Department of Child Health, Faculty of Medicine, University of Tsukuba, Tsukuba, Japan; 6 Structural Medicine, United Graduate School of Drug Discovery and Medical Information Science, Gifu University, Gifu, Japan; 7 Department of Pediatrics and Virology, St Mary’s Medical School Bldg, Imperial College London, London, UK; 8 Imagine Institute, Paris University, Paris, France; 9 Study Center for Primary Immunodeficiencies, Assistance Publique des Hôpitaux de Paris (APHP), Necker-Enfants Malades University Hospital, Paris, France; 10 University Paris Descartes Sorbonne Paris Cité, Paris, France; 11 Oxford Autoimmune Neurology Group, Nuffield Department of Clinical Neurosciences, University of Oxford, Oxford, UK; 12 Department of Applied Genomics, Kazusa DNA Research Institute, Kisarazu, Japan; 13 St. Giles Laboratory of Human Genetics of Infectious Diseases, Rockefeller Branch, Rockefeller University, New York, NY USA; 14 Howard Hughes Medical Institute, New York, NY USA; 15 Present Address: Japan Red Cross, Chugoku-Shikoku Block Blood Center, Hiroshima, Japan

**Keywords:** anti-NMDAR encephalitis, autoimmunity, HHV6, IRAK4

## Abstract

IRAK4 deficiency is an inborn error of immunity predisposing patients to invasive pyogenic infections. Currently, there is no established simple assay that enables precise characterization of *IRAK4* mutant alleles in isolation. Anti-N-methyl-D-aspartate receptor (NMDAR) encephalitis is an autoimmune condition that is characterized by psychiatric symptoms, involuntary movement, seizures, autonomic dysfunction, and central hypoventilation. It typically occurs in adult females associated with tumors. Only a few infantile cases with anti-NMDAR encephalitis have been so far reported. We identified a 10-month-old boy with IRAK4 deficiency presenting with anti-NMDAR encephalitis and human herpes virus 6 (HHV6) reactivation. The diagnosis of IRAK4 deficiency was confirmed by the identification of compound heterozygous mutations c.29_30delAT (p.Y10Cfs*9) and c.35G>C (p.R12P) in the *IRAK4* gene, low levels of IRAK4 protein expression in peripheral blood, and defective fibroblastic cell responses to TLR and IL-1 (TIR) agonist. We established a novel NF-κB reporter assay using IRAK4-null HEK293T, which enabled the precise evaluation of *IRAK4* mutations. Using this system, we confirmed that both novel mutations identified in the patient are deleterious. Our study provides a new simple and reliable method to analyze *IRAK4* mutant alleles. It also suggests the possible link between inborn errors of immunity and early onset anti-NMDAR encephalitis.

**Electronic supplementary material:**

The online version of this article (10.1007/s10875-020-00885-5) contains supplementary material, which is available to authorized users.

## Introduction

Toll-like receptors (TLRs) sense microbial products and play an important role in innate immunity [1]. Activation of the TLR response results in increased production of inflammatory cytokines such as IL-6 and type I interferons, a key component of the anti-viral state, and secretion of chemokines to attract innate immune cells [2]. Autosomal recessive (AR) interleukin-1 receptor (IL-1R)-associated kinase 4 (IRAK4) deficiency, together with myeloid differentiation primary response gene 88 (MyD88) deficiency, is a primary immune deficiency that impairs IL-1R and TLR family signaling [3]. Patients with AR IRAK4 deficiency show a predominant susceptibility to invasive infections with pyogenic bacteria such as *Streptococcus pneumoniae*, *Staphylococcus aureus*, and *Pseudomonas aeruginosa* in early childhood, with severe and often fatal outcomes [4, 5]. However, no deaths have been reported after 8 years of age, which is likely due to the acquisition of humoral immunity and immunologic memory [4]. Prophylactic antibiotic treatment, vaccinations against pyogenic bacteria, and intravenous immunoglobulin (IVIG) starting early in life are recommended as prophylactic treatments [1]. The diagnosis of IRAK4 deficiency is performed by identification of biallelic mutations in the *IRAK4* gene and cellular assay by testing cytokine production to TLR and IL-1R ligands using a patient’s peripheral blood and fibroblasts. However, cells and patients with inherited MyD88 deficiency are indistinguishable from cells and patients with inherited IRAK4 deficiency [4]. Furthermore, there is no established simple assay that enables a precise characterization of rare *IRAK4* variants identified in isolation.

Anti-N-methyl-D-aspartate receptor (NMDAR) encephalitis is an autoimmune encephalitis characterized by psychiatric symptoms, involuntary movement, seizures, autonomic dysfunction, and central hypoventilation. Anti-NMDAR encephalitis was originally described in 2007 as a potentially reversible immune-mediated paraneoplastic disorder in a young woman with ovarian teratomas [6]. Only a few cases have been reported in the literature in childhood and infants cases although nearly 40% of patients with anti-NMDAR encephalitis are under 18 years of age [7, 8]. The precise mechanism of anti-NMDAR antibody production in children still remains unclear. Multiple studies have shown that early treatment of anti-NMDAR encephalitis in children leads to better outcomes [9]. In the current study, we report a rare case of IRAK4 deficiency presenting with severe neurological sequelae associated with anti-NMDAR encephalitis and human herpes virus 6 (HHV6) reactivation. We newly established a NF-κB reporter assay system that enabled precise evaluation of *IRAK4* mutations. Using this system, we confirmed that two novel mutations in *IRAK4* identified in the patient are deleterious.

## Material and Methods

### Case Report

The patient is a 10-month-old Japanese boy and the first child of nonconsanguineous parents. He was born after an uneventful pregnancy and his delivery was normal. There was no family history of neurological or metabolic disorders or immunodeficiency disease. The patient had a history of delayed separation of the umbilical cord. His development was normal and he was fully immunized on schedule until 10 months of age having received three doses each of the diphtheria, tetanus toxoids and acellular pertussis vaccine (DPT), *Haemophilus influenzae* type B (Hib) vaccine, and 13-Valent Pneumococcal Conjugate Vaccine (PCV13), and one Bacilli Calmette-Guérin (BCG) vaccine. Although the patient suffered from otitis media, he had no history of severe invasive bacterial infection. He had a febrile episode that was clinically diagnosed as typical exanthema subitum at the age of 8 months, approximately 2 months before the onset of anti-NMDAR encephalitis. He also had a self-limiting febrile episode 4 weeks before the onset. At the age of 10 months, he was admitted to the local hospital due to a fever of 38.4 degrees and dyspnea. A C-reactive protein (CRP) test was normal. He had a convulsion which was interrupted by midazolam (designated as day 1). However, he was required mechanical ventilation for severe respiratory dysfunction after this episode. He also needed continuous intravenous infusion of midazolam not only for sedation, but also to inhibit generalized convulsion. The cerebrospinal fluid (CSF) analysis was clear and negative cultures for any microorganisms without pleocytosis (Table 1). No virus was detected in CSF, throat, or stool. Magnetic resonance imaging (MRI) performed on admission showed no abnormal findings. The absence of anti-NMDAR antibodies in sera obtained at this time was confirmed in a later analysis (the result obtained at day 74). He was weaned off mechanical ventilation on day 9. However, central hypoventilation, which requires respiratory support with a high-flow nasal cannula, became apparent after mechanical ventilation was stopped. He had a fever of 38.2 degrees again on day 19. HHV6 was isolated by virus culture from blood, stool, and throat specimens, which strongly suggested the presence of HHV6 reactivation. After that, his symptoms of central hypoventilation worsened. He was transferred to our hospital on day 31 because of the progression of his neurological symptoms.

**Table 1:** Cerebrospinal fluid examination results

	Day 1	Day 30	Day 32	Day 204	Reference range
Cell count (/μl)	5	64	37	2	0–20
Differential count (%)					
Neutrophils	20	2	5	0	
Lymphocytes	80	98	95	100	
Monocytes and others	0	0	0	0	
Protein (mg/dl)	21	91	65	24	15–45
IgG index	NA	NA	1.63	0.54	< 0.73
Glucose (mg/dl)	76	59	64	58	50–80
Lactic acid (mg/dl)	NA	9.7	12.3	NA	3.7–16.3
Pyruvic acid (mg/dl)	NA	0.5	0.87	NA	0.30–0.90
Oligoclonal band	NA	NA	Positive	Negative	
Myelin basic protein (pg/ml)	NA	NA	< 31.3	< 31.3	0–102.0
Anti-NMDA-receptor antibodies	NA	NA	Positive (1:20)	Negative	

**Fig. 1:**
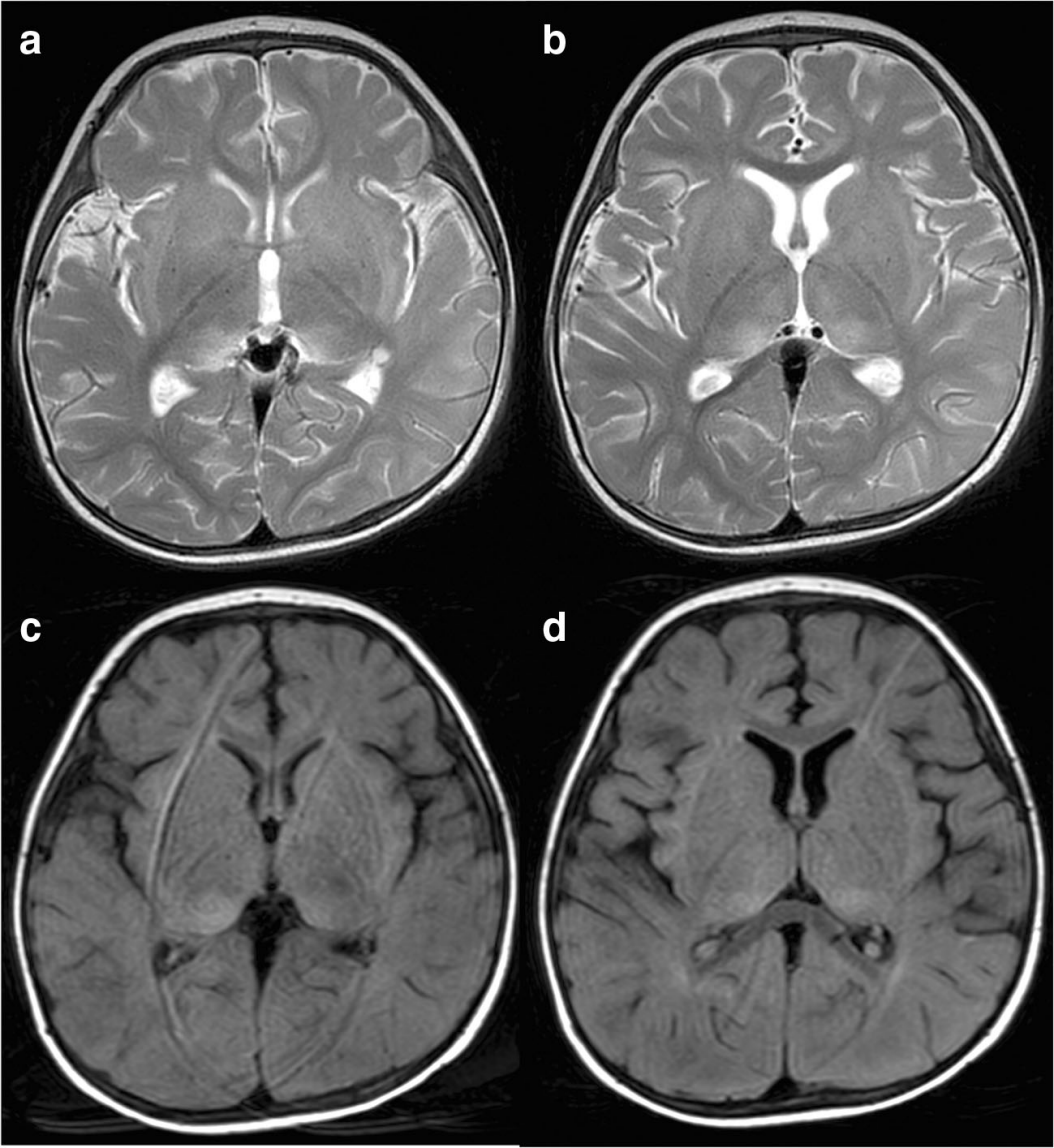
Brain MRI obtained at day 4. **a, b** The axial T2 weighted image (T2WI) showed high intensity regions in the bilateral thalamus. **c, d** The axial fluid attenuation inversion recovery (FLAIR) image showed high intensity regions in the bilateral thalamus

At the time of admission to our hospital, his consciousness was impaired. He did not maintain eye contact and his muscular tonus was weak. He needed the support of non-invasive positive pressure ventilation for dyspnea. CSF examination showed pleocytosis (37 leukocytes/mm^3^, reference range (RR) 0–30) with predominant lymphocytosis (lymphocyte 95%), high protein level (65 mg/dl, RR 15–45), elevated IgG index (1.63, RR 0.33–0.63), and the absence of oligoclonal IgG bands with normal glucose level (64 mg/dl, RR 50–80) (Table 1). The CSF bacterial and virus culture was negative. No viruses, including herpes simplex virus (HSV), cytomegalovirus (CMV), or Epstein-Barr virus (EBV), were isolated by PCR from CSF. However, we identified anti-NMDA-receptor antibodies (1:20, RR < 1:1) in CSF (Table 1) in a later analysis (results obtained 43 days after the examination). Brain MRI revealed hyperintensities in the bilateral thalami in a T2 weighted imaging (T2WI) (Fig. 1a,b) and fluid attenuation inversion recovery (FLAIR) imaging (Fig. 1c,d). In contrast, spinal MRI detected no remarkable findings. EEG revealed diffuse slowing. Nerve conduction studies (NCS), short-latency somatosensory evoked potentials (SSEP), and auditory brainstem responses (ABR) showed no abnormal findings. No solid tumors were detected by whole body CT scanning.

After admission, the deterioration of motor and psychiatric functions was exacerbated. The choreoathetosis of his limbs and trunk and orolingual-facial dyskinesias had worsened with no social contact, and he needed nasogastric tube feeding. His involuntary movements were treated with haloperidol and trihexyphenidyl hydrochloride. The patient was suspected to have autoimmune encephalitis and started treatment with methylprednisolone pulse therapy (30 mg/kg for 3 days) combined with IVIG (1 g/kg for 2 days) from day 32. Oral prednisolone (0.5 mg/kg/day) was subsequently administered following methylprednisolone therapy. One month later, his involuntary movement was dramatically diminished, and he was able to sit alone and to take milk and food by mouth. At this time, based on the detection of anti-NMDAR antibodies in CSF, the patient was diagnosed with anti-NMDAR encephalitis. Oral administration of prednisolone continued for 6 months (Table 1). Administration of haloperidol and trihexyphenidyl hydrochloride for involuntary movement continued for 5 months. He was discharged from our hospital on day 82.

Compound heterozygous mutations c.29_30delAT (p.Y10Cfs*9) and c.35G>C (p.R12P) in the *IRAK4* gene were identified by whole exome sequencing at 12 months of age. The lack of IRAK4-mediated TLR signaling was confirmed by the analysis of the patient’s peripheral blood and fibroblasts. He was thus given a diagnosis of IRAK4 deficiency. Prophylaxis treatment with oral amoxicillin was initiated immediately after the diagnosis of IRAK4 deficiency, and prophylactic IVIG was started at 27 months of age. A follow-up CSF analysis performed at 15 months of age was negative for anti-NMDAR antibodies (Table 1). Obstructive sleep apnea was identified by polysomnography at 16 months. The B cell immunophenotyping which was performed at 19 months detected no obvious abnormality in the absolute number of B cells and the frequency of naïve, non-switched and switched B cells. The frequency of transitional B cells and plasmablast was also normal. The HLA genotype of the patient was HLA-A*02:01, 33:03, HLA-B*13:01, 44:03, HLA-C*03:04, 14:03, and HLA-DRB1*12:02, 13:02. At 19 months, 9 months after the initial onset of symptoms, he could walk with support. No involuntary movement was noted; however, he had not begun talking at this time. At 36 months, he began to take aripiprazole for his irritability, and his psychomotor development was equivalent to an 18-month old. Although he has previously suffered from mild otitis media, he has not had a history of severe invasive bacterial infection with prophylactic treatments.

### Molecular Genetics

Genetic tests were performed after the written informed consent of the participants or their parents was obtained. This study was approved by the Ethics Committees and Internal Review Boards of Hiroshima University. Genomic DNA was extracted from peripheral blood leukocytes and subjected to whole exome sequence (WES) and/or Sanger sequencing. The detailed WES method was described previously [10]. We used the pcDNA3+ expression vectors that contain N-terminal FLAG-tagged wild-type (WT) or mutant (p.R12C, p.R20W, or p.Q293*) *IRAK4* genes [11]. We generated expression vectors encoding p.R12P and p.Y10Cfs*9 mutant *IRAK4* with PCR-based mutagenesis of the pcDNA3+ WT IRAK4 vector with mismatched PCR primers. The primer sequences and PCR conditions are available upon request.

Detailed methods of quantitative real-time PCR, reverse transcription PCR (RT-PCR), flow cytometry, immunoblot analysis, and TLR testing of patient fibroblasts are shown in previous reports and the Supplementary Electronic Materials and methods [12, 13].

### IRAK4-Deficient Cell Preparation

IRAK4-deficient HEK293 cells were created using the CRISPR/Cas9 system. HEK293 cells (purchased from the Japanese Collection of Research Bioresources, Osaka, Japan) were transfected with the IRAK4 CRISPR/Cas9 KO plasmid (h): sc-416405 by Nucleofector II and the Cell Line Nucleofector kit V (Lonza, Basel, Switzerland) using the Q-001 program. Single cell clones adjusted by the limited dilution method were then cultured. Successful *IRAK4* knockout was verified by the detection of a DNA fragment of the target site and the direct sequencing of genomic DNA from candidate clones along with the detection of endogenous protein expression with an immunoblot.

### Luciferase Reporter Assay

IRAK4-null HEK293 cells were transfected with pcDNA3.1+FLAG-IRAK4 WT or mutant IRAK4 alleles, IL-18RAcPL, Igkcona-Luc (provided by S. Yamaoka), and pRL-TK (Promega, Madison, Wisconsin, USA) using lipofectamine LTX according to the manufacturer’s protocol. They were then stimulated with recombinant IL-18 (50 ng/ml) created using a previously described method for 6 h [14]. Luciferase reporter gene activities were analyzed using the Dual-Luciferase Reporter Assay System (Promega). The experiments were performed in triplicate, and the data are expressed in relative luciferase units (RLU). Three independent experiments were performed to confirm the results.

### Protein Structure Analysis

The ternary structure of the death domain complex of MyD88, IRAK4, and IRAK2 (PDB code: 3MOP) was used as a template [15]. The structures of the mutant IRAK4 proteins were built with the MOE software (Molecular Operating Environment 2013.08; Chemical Computing Group Inc., Montreal, Canada, 2013; www.chemcomp.com).

### Detection of Anti-NMDAR Antibody in Patients with IRAK4 or MyD88 Deficiency

The detection of anti-NMDAR antibodies was performed by live cell-based assay as previously described [16].

## Results

### Identification of IRAK4 Mutations in the IRAK4 Gene

**Fig. 2:**
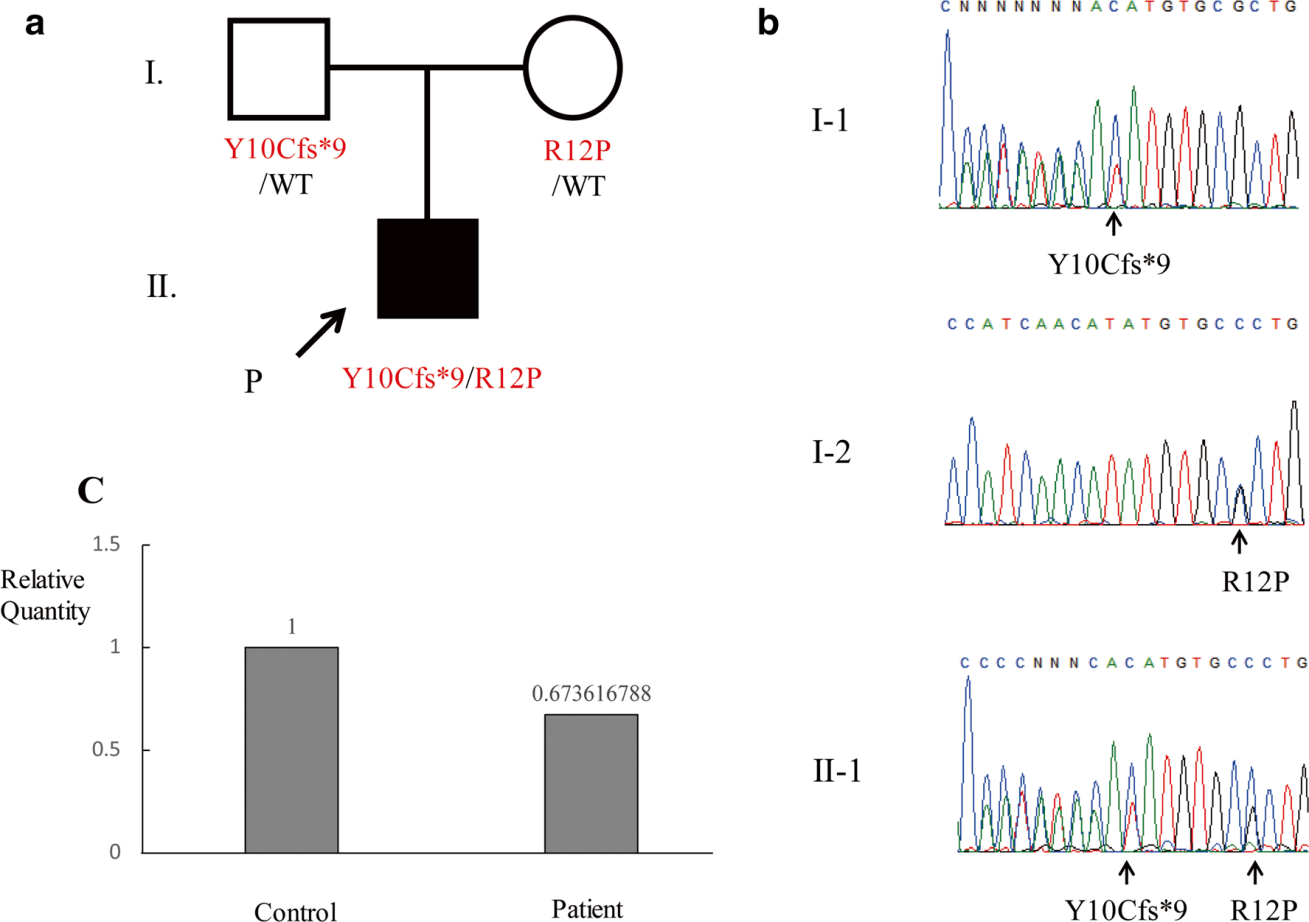
Identification of *IRAK4* mutations and detection of *IRAK4* mRNA expression in PBMCs. **a, b** Familial segregation of *IRAK4* mutations. The novel compound heterozygous mutation in the *IRAK4* gene was detected in the patient (II.1). The Y10Cfs*9 and R12P mutations were inherited from his asymptomatic father and mother, respectively. **c** The expression of the *IRAK4* mRNA was assessed by RT-qPCR from PBMCs of the patient and one healthy control. *IRAK4* mRNA from the patient was about two-thirds lower than that of the healthy control

As the development of anti-NMDAR encephalitis in infantile periods is quite rare, we suspected the presence of a genetic background in this patient. We thus performed a comprehensive and unbiased genetic study using WES. After the filtering process, several rare variations were annotated in genes that are reported to be related to inborn error of immunity (Table S1) [17]. Among them, rare variants in *EPG5*, *STK4*, *C5*, and *C8A* were unlikely to be disease causing due to their inheritance patterns, clinical phenotypes, and laboratory data that showed normal complement levels. The variations in the *IRAK4* gene, p.Y10Cfs*9, and p.R12P were confirmed by Sanger sequencing and were considered to be the best candidate variation in the list of the annotated variations. No other candidate rare variants that could explain the patient’s manifestations were identified by WES. The p.Y10Cfs*9 and p.R12P variations were inherited from his father and mother, respectively (Fig. 2a and b). Neither of the variations were found in the Single Nucleotide Polymorphism Database (dbSNP), 1000 Genome Projects, Exome Aggregation Consortium (ExAc) database, or Genome Aggregation Database (gnomAD). We performed RT-qPCR to analyze *IRAK4* mRNA expression in the patient’s peripheral blood mononuclear cells (PBMCs). The expression of *IRAK4* mRNA in the patient was approximately two-thirds compared with a healthy subject (Fig. 2c). The expression of *IRAK4* mRNA in the patient was confirmed by RT-PCR (Fig. S2A). Sanger sequencing of RT-PCR product revealed that both p.Y10Cfs*9 and p.R12P allele almost equally expressed in mRNA level (Fig. S2B).

### TNF-α Production and IRAK4 Protein Expression

**Fig. 3:**
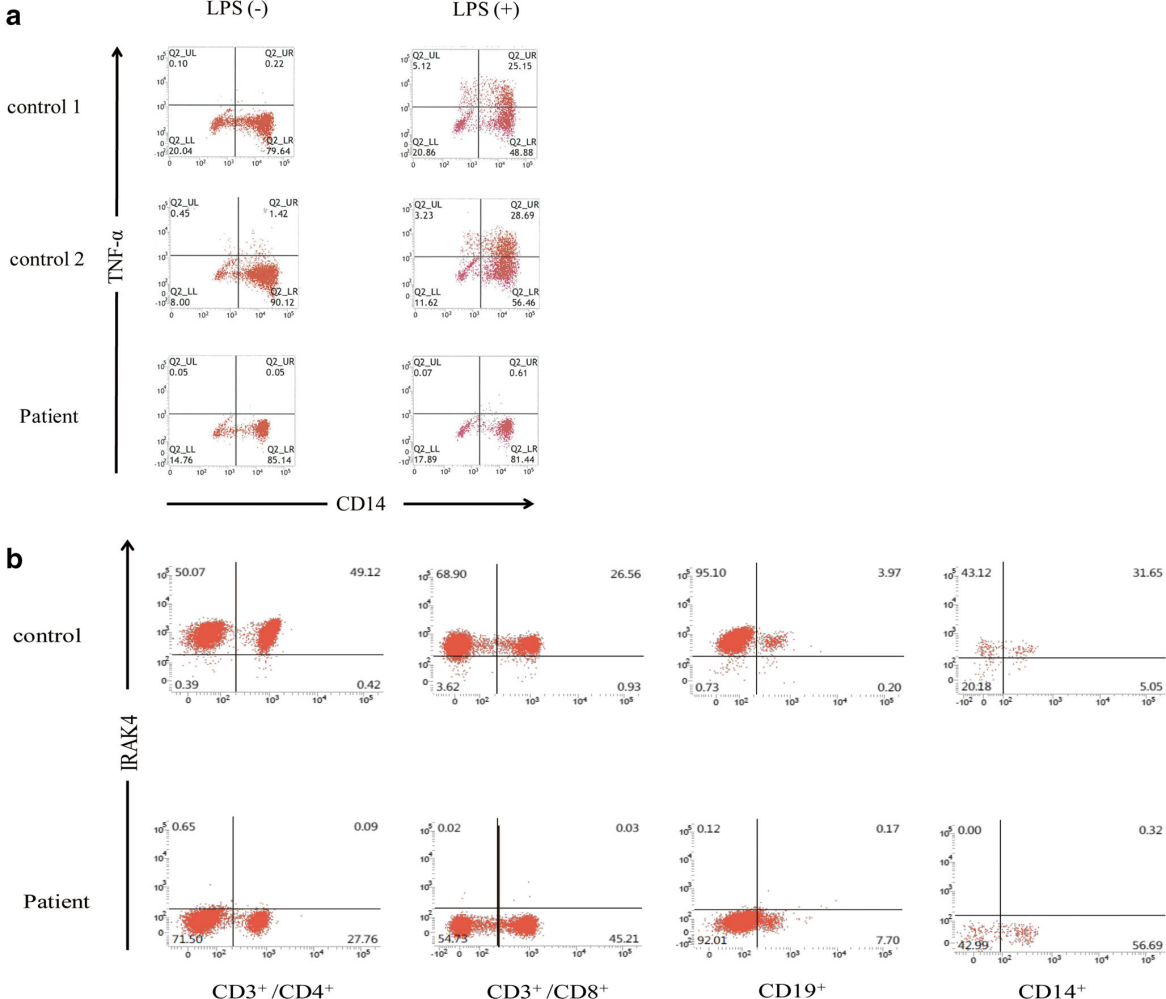
Flow-cytometric analysis of TNF-α production and IRAK4 protein levels of PMBCs. **a** Flow cytometric analysis of intracellular TNF-α production of monocytes in response to LPS. The patient’s CD14^+^ monocytes display impaired TNF-α production in response to LPS stimulation. **b** Flow cytometric analysis of IRAK4 protein expression. IRAK4 expression was abolished in CD3^+^ /CD4^+^ T cells, CD3^+^ /CD8^+^ T cells, CD19^+^ B cells, and CD14^+^ monocytes

PBMCs from the patient were stimulated with LPS, and intracellular TNF-α production was examined by the intracellular staining of TNF-α according to a previous report [18]. As shown in Fig. 3a, TNF-α production stimulated by LPS treatment of the patient’s CD14^+^ cells was significantly impaired compared with that of healthy subjects. IRAK4 positive cells were low among in CD3^+^/CD4^+^, CD3^+^/CD8^+^, CD19^+^, and CD14^+^ cells (Fig. 3b).

### IL-6 Production with the Stimulation of Fibroblasts with Various TLR Ligands

**Fig. 4:**
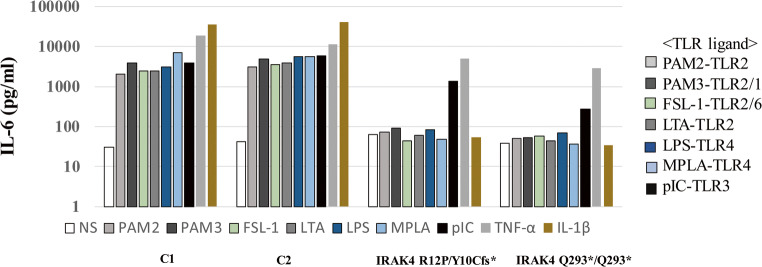
IL-6 production with the stimulation of various TLR ligands in fibroblasts. IL-6 production by SV40-immortalized fibroblasts from healthy controls and two IRAK4-deficient patients after 24 h of stimulation with various TLR agonists. IL-6 production was defective with the stimulation of TLR1, TLR2, TLR4, and TLR6 but not of TLR3 in cells expressing R12P/Y10Cfs* and Q293*/Q293* alleles

IL-6 production was tested by the stimulation with TLR ligands, such as PAM2 (PAM2CSK4, a TLR2/6 agonist), PAM3 (PAM3CSK4, a TLR1/2 agonist), FSL1 (a TLR 1/2 agonist), LTA (a TLR2 agonist), LPS (a TLR4 agonist), MPLA (a TLR4 agonist), poly(I:C), TNF-α, and IL-1β using SV40-immortalized fibroblasts. As shown in Fig. 4, defective responses to PAM2, PAM3, FSL1, LTA, LPS, MPLA, and IL-1β are detected in the SV40 fibroblasts from the patient, as well as a disease control with Q293X homozygous *IRAK4* mutation [5]. In contrast, normal IL-6 production in response to TNF-α was observed in the patient’s SV40 fibroblasts. The response of the patient’s fibroblasts to poly(I:C) was slightly decreased compared with that of wild-type cells.

### NF-κB Reporter Assay to Evaluate Function of IRAK4 Mutant Alleles

**Fig. 5:**
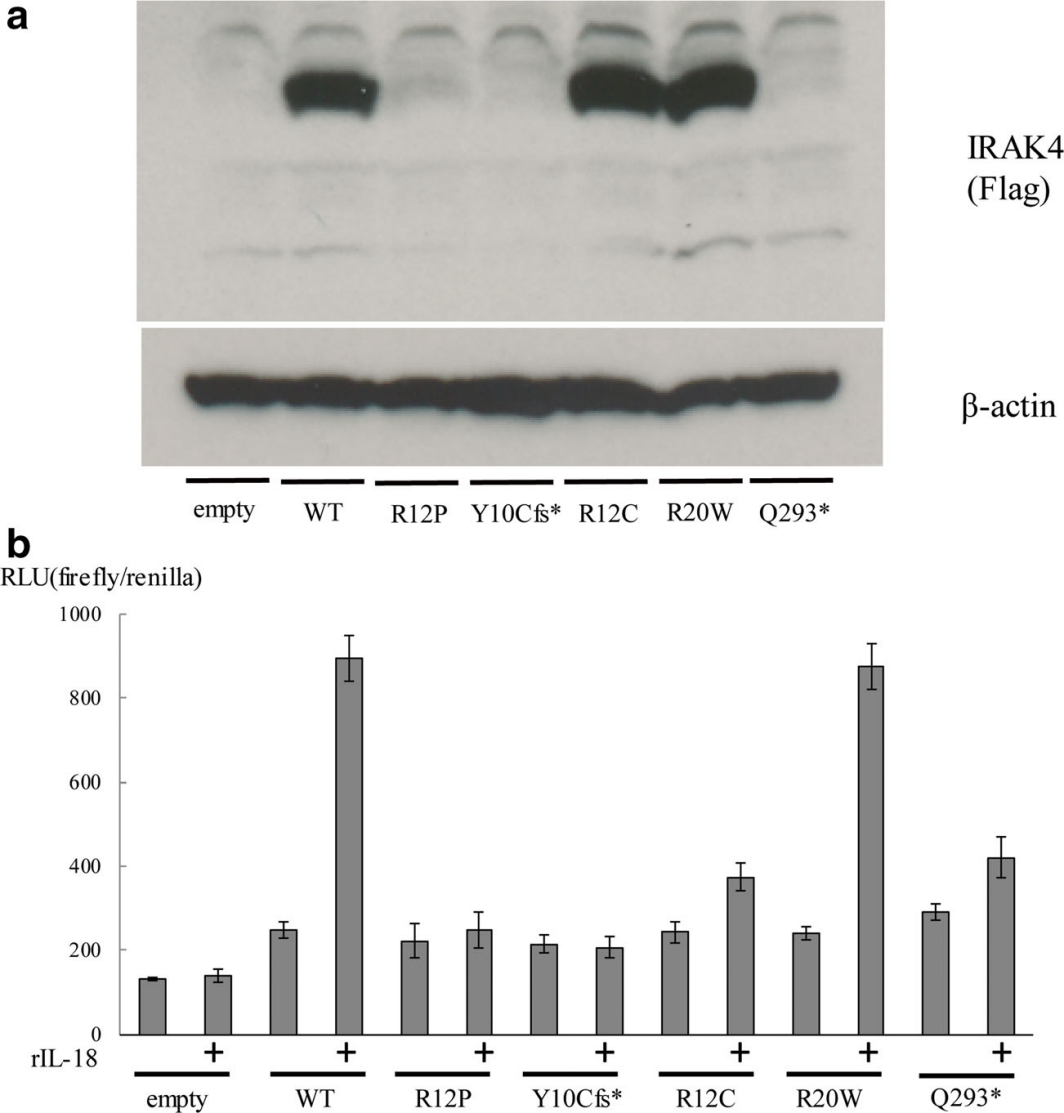
IRAK4 protein expression and IL-18-induced IRAK4-mediated NF-κB activation. **a** IRAK4 and β-actin protein levels in HEK293T transfectants. Both Y10Cfs* and Q293* mutations completely abolished IRAK4 protein expression. The R12P mutation severely impaired IRAK4 protein expression, whereas the protein expression was normally observed in the R12C mutation. The IRAK4 expression of the R20W polymorphism was comparable with that of WT. **b** NF-κB reporter activity in HEK293T transfectants. The R12P, Y10Cfs*, R12C, and Q293* (reported previously) mutant alleles showed severe impairment in IL-18-induced NF-κB activation. The R20W polymorphism showed equivalent levels of IL-18-induced NF-κB activation to WT IRAK4

The diagnosis of IRAK4 deficiency is usually confirmed by cellular assay using a patient’s PBMCs or fibroblasts. However, there is no established simple assay system that enables precise evaluation of *IRAK4* mutations in isolation. We transiently transfected HEK293T cells with an empty vector or with plasmids encoding *IRAK4* p.R12C, a missense mutation [19], p.Q293*, the most common mutation found in Europe [4], or a single nucleotide polymorphism (p.R20W) as well as the *IRAK4* variants p.Y10Cfs* or p.R12P, identified in our patient. We then evaluated the IRAK4 expression by immunoblotting. As shown in Fig. 5a, p.Y10Cfs* completely abolished protein expression similarly to the previously reported p.Q293* *IRAK4* mutation [3]. The p.R12P variation severely impaired IRAK4 protein expression, whereas protein expression was normal for the p.R12C mutation that affects the same amino acid. Further, expression of the IRAK4 p.R20W variant was comparable with that of WT. The immunoblot results were consistent with the flow cytometry results, which showed few IRAK4 positive populations in the patient’s PBMCs.

Next, we evaluated the functional significance of *IRAK4* variants with a NF-κB reporter assay. Yamamoto et al. previously developed a NF-κB reporter assay system to assess the functional significance of *IRAK4* variants [11]. However, in this system, WT IRAK4 decreased IL-18-induced NF-κB activation. This assay successfully segregated WT IRAK4 from four mutants (c.118insA, p.R183*, p.Q293*, and p.G298D), but it failed to show molecular defects due to the p.R12C mutant. To resolve these problems, we generated IRAK4-null HEK293T cell lines for use in the NF-κB reporter assay. As shown in Fig. 5b, WT IRAK4 increased NF-κB activation in IL-18-treated cells. NF-κB activation was abolished by p.R12P and p.Y10Cfs* variations, displaying lower activity than the p.Q293* and R12C mutations. By contrast, the p.R20W polymorphism showed a comparable level of NF-κB activations with WT. Taken together, we succeeded in establishing a NF-κB reporter assay system that could be used to precisely evaluate the functional significance of *IRAK4* variations. Both p.Y10Cfs* and p.R12P mutations abolish IRAK4-mediated NF-κB activation by IL-18.

### Prediction of the Mutational Effect of the IRAK4 Gene

The R12 residue is located on the surface of the IRAK4 death domain that mediates interaction with MyD88 [11]. In silico analysis was used to study the effect of IRAK4 R12C and R12P substitutions on the interaction with MyD88 (Fig. S2). IRAK4 R12C expression was previously reported to be preserved by the interaction with MyD88 [11]. By contrast, in silico analysis suggested that unlike R12C, R12P does not create a stabilizing internal hydrogen bond and fails to interact with MyD88 (Fig. S2). The in silico study is thus consistent with and may explain the results of our in vitro reporter study that showed severely impaired function and expression of the patient’s mutated R12P IRAK4 protein (Fig. 5a).

### Antibody Prevalence in Other IRAK4-Deficient Patients

In the current study, the presence of anti-NMDAR antibodies was identified in CSF during the episode of encephalitis. However, a follow-up CSF study performed 5 months later was negative for anti-NMDAR antibodies. We next investigated whether other patients with IRAK4 or MyD88 deficiency have anti-NMDAR antibodies. We measured anti-NMDAR antibodies in sera from patients with IRAK4 (*n* = 5) or MyD88 (*n* = 1) deficiency. However, none of the patients had anti-NMDAR antibodies.

## Discussion

The case presented herein demonstrated IRAK4 deficiency with anti-NMDAR encephalitis and HHV6 reactivation. IRAK4 deficiency was determined by the identification of compound heterozygous mutations in the *IRAK4* gene, low levels of protein expression for IRAK4 in CD14-positive cells, defective production of TNF-α in CD14-positive cells, and defective NF-κB activation by IL-18 stimulation in *IRAK4-*null cells expressing the patient’s alleles. To date, 24 mutations have been identified in patients with IRAK4 deficiency (Table S2) [3, 4, 11, 18–29]. Among them, 20 mutations are nonsense, frameshift, or splice site mutations that are expected to abrogate their functions. However, four mutations are nonsynonymous which require experimental verification to confirm their pathogenicity. The existing NF-κB reporter assay system can potentially misevaluate the pathogenesis of IRAK4 mutants [11]. Moreover, the assay shows that WT IRAK4 has a negative impact on IL-18-induced NF-κB activation, although IL-18 upregulates NF-κB via IRAK4 in general. To resolve these problems, we succeeded in establishing a precise assay using CRISPR-generated *IRAK4*-deficient HEK293 cells. To the best of our knowledge, it is the first in vitro assay system that reproduces IL-18-induced WT IRAK4-mediated NF-κB activation, enabling us to distinguish pathogenic mutations, including p.R12C, not only from WT, but also from the known p.R20W allele. This assay system confirmed that two novel mutations, p.Y10Cfs* and p.R12P, identified in the patient are deleterious. Recent progress in comprehensive genetic studies enabled us to detect pathogenic mutations in previously undiagnosed patients. At the same time, such studies rapidly increased the identification of rare variants that need functional characterization to evaluate their pathogenicity. The evaluation of TNF-α production by flow cytometry is a rapid and reliable functional test to confirm the pathogenesis of rare variants found in IRAK4. However, it requires viable peripheral blood from the patients. Especially under the limited availability of viable patients’ samples, the NF-κB reporter assay system that we established in the current study could be a simple and a reasonable tool to evaluate uncharacterized rare variants in the *IRAK4* gene.

Picard et al. summarized clinical features and outcomes of 49 patients with IRAK4 deficiency and 22 with MyD88 deficiency [1]. The initial infectious phenotypes of the majority of the patients with IRAK4 deficiency were severe bacterial infections, such as *S. pneumoniae*, *S. aureus*, and rarely *P. aeruginosa* and *Shigella sonnei*. However, IRAK4-deficient patients were not particularly susceptible to most microorganisms, including viruses, parasites, and fungi. In contrast, curiously, MyD88- and IRAK4-deficient mice show susceptibility to viruses, including HHV1 and HHV2 [30]. It is noteworthy that rare neurological findings associated with anti-NMDAR encephalitis and/or HHV6 reactivation in an infant led us to study whole exome sequencing, resulting in the identification of novel compound heterozygous mutations in the *IRAK4* gene. The coexistence of anti-NMDAR encephalitis and HHV6 reactivation in this patient may reveal an unknown manifestation associated with IRAK4 deficiency. It is well known that anti-NMDAR encephalitis is triggered by HSV-1 infection [31–35]. The post-infectious autoimmune process that follows the HSV-induced brain damage is thought to be the cause of anti-NMDAR encephalitis [36, 37]. HHV6 is a neurotropic DNA virus that establishes chronic latency in brain tissue [38]. We suspect that HHV6 reactivation induced some brain damage or dysregulation of host immunity that triggered anti-NMDAR antibody production. The limitation of our study is a lack of direct evidence that demonstrates the relationship between IRAK4 deficiency and development of anti-NMDAR encephalitis and/or HHV6 reactivation. To date, no cases with anti-NMDAR encephalitis or severe virus infections have been reported in IRAK4-deficient patients. Although we investigated patients with IRAK4 (*n* = 5) or MyD88 (*n* = 1) deficiency, no patients had anti-NMDAR antibodies in sera. Further accumulations of cases are necessary to fully characterize the association of these rare clinical manifestations in patients with IRAK4 deficiency.

TLRs are a key family of pattern recognition receptors (PRRs) involved in driving autoimmune inflammation. The inhibitors of TLR binding or signaling have been applied to potential therapeutic agents for autoimmune and other inflammatory diseases [39, 40]. While patients with IRAK4 deficiency accumulate autoreactive B cells in the blood, the inhibition of the TLR signaling pathway is unlikely to develop autoimmune disorders [41]. Defective TLR signaling, especially that of TLR7 and TLR9, appears to inhibit activation of these autoreactive B cells, as shown in animal models [42]. Collectively, the production of autoantibodies is theoretically suppressed in patients with IRAK4 deficiency irrespective of the presence of large numbers of autoreactive B cells. However, Hugle reported a case of a patient with antinuclear antibody (ANA)-positive juvenile idiopathic arthritis with genetically confirmed IRAK4 deficiency [43]. The presence of the previous case, together with the case presented in the current study, suggest that autoimmune diseases can occur in patients with IRAK4 deficiency in conflict with the paradigm of IRAK4-mediated signaling being critically necessary for the development of reactive autoantibodies and autoimmune diseases.

In most patients with IRAK4 deficiency, the first bacterial infection occurs before the age of 2 years. Patients are highly susceptible to life-threatening invasive bacterial diseases caused by *Streptococcus pneumoniae* and *Staphylococcus aureus*. Delayed diagnosis and inappropriate treatment of patients with IRAK4 deficiency may not only lead to fatal invasive infection but also to irreversible organ damage later in life [44]. Prophylactic treatments such as antibiotic prophylaxis, immunization by vaccines, and IVIG have been significantly effective to avoid invasive bacterial infections in such patients. Thus, early accurate diagnosis of IRAK4 deficiency is important to achieve life-saving treatment. Our patient’s atypical clinical manifestation and development of anti-NMDAR encephalitis in infancy led us to sequence his whole exome and identify IRAK4 deficiency. This enabled us to start anti-bacterial prophylaxis before severe bacterial infections could develop. Indeed, the patient has not experienced severe bacterial infections in the first 4 years of his life owing to such prophylactic treatments. The current case also revealed the possibility that genetic studies can contribute to characterizing infantile cases with anti-NMDAR encephalitis. Further accumulation of cases and characterization of the molecular pathogenesis of IRAK4 deficiency are expected to elucidate the risk of viral infections and/or anti-NMDAR encephalitis in patients with IRAK4 deficiency.

## Electronic Supplementary Material

ESM 1 (DOCX 403 kb).
