# Supplementary material for: IRAK4 Deficiency Presenting with Anti-NMDAR Encephalitis and HHV6 Reactivation
Source: J Clin Immunol. 2020 Oct 20;41(1):125–35. doi: 10.1007/s10875-020-00885-5 (PMC7846526; doi:10.1007/s10875-020-00885-5)
Supplement: Supplementary file 1 — (DOCX 403 kb). [file 10875_2020_885_MOESM1_ESM.docx]

**Electronic Supplementary Material**

**IRAK4 deficiency presenting with anti-NMDAR encephalitis and HHV6 reactivation**

**Authors**

Shiho Nishimura^1, §^, Yoshiyuki Kobayashi^1, §^, Hidenori Ohnishi^2^, Kunihiko Moriya^3, 4Φ^, Miyuki Tsumura^1^, Sonoko Sakata^1^, Yoko Mizoguchi^1^, Hidetoshi Takada^5^, Zenichiro Kato^2, 6^, Vanessa Sancho-Shimizu^7^, Capucine Picard^8, 9, 10^, Sarosh R Irani^11^, Osamu Ohara^12^, Jean-Laurent Casanova^3, 10, 13, 14^, Anne Puel^3, 10, 13^, Nobutsune Ishikawa^1^,

Satoshi Okada^1^, Masao Kobayashi^1, 15Φ^

**Institutions**

^1^Department of Pediatrics, Hiroshima University Graduate School of Biomedical and Health Science, Hiroshima, Japan

^2^Department of Pediatrics, Graduate school of Medicine, Gifu University, Gifu, Japan

^3^Laboratory of Human Genetics of Infectious Diseases, Necker Branch, INSERM UMR 1163, Paris, France

^4^Department of Pediatrics, Tohoku University Graduate School of Medicine, Sendai, Japan

^5^Department of Child Health, Faculty of Medicine, University of Tsukuba, Tsukuba, Japan

^6^Structural Medicine, United Graduate School of Drug Discovery and Medical Information Science, Gifu University, Gifu, Japan

^7^Department of Pediatrics and Virology, St Mary’s Medical School Bldg, Imperial College London, London, UK

^8^Imagine Institute, Paris University, Paris, France

^9^Study center for primary immunodeficiencies, Assistance Publique des Hôpitaux de Paris (APHP), Necker-Enfants Malades University Hospital, Paris, France

^10^University Paris Descartes Sorbonne Paris Cité, Paris, France

^11^Oxford Autoimmune Neurology Group, Nuffield Department of Clinical Neurosciences, University of Oxford, UK

^12^Department of Applied Genomics, Kazusa DNA Research Institute, Kisarazu, Japan

^13^St. Giles Laboratory of Human Genetics of Infectious Diseases, Rockefeller Branch, Rockefeller University, New York, NY, United States

^14^Howard Hughes Medical Institute, New York, USA

^15^Japan Red Cross, Chugoku-Shikoku Block Blood Center, Hiroshima, Japan

^Φ^current affiliation

^§^equal contributions

**Corresponding Author**Satoshi Okada, MD, PhD

E-mail: sokada@hiroshima-u.ac.jp

**This file includes:**

**Supplemental Tables 1, 2**

**Supplemental Figures 1, 2**

**Supplemental material and methods**

**Table S1.** Summary of candidate genes by whole exome sequencing

| **Gene** |  | dbSNP | ExAC_  ALL | gnomAD_ALL | HGVS.c | HGVS.p |
| --- | --- | --- | --- | --- | --- | --- |
| IRAK4 | Hetero | ・ | ・ | ・ | c. 29_30delAT | p. Tyr10fs |
| IRAK4 | Hetero | ・ | ・ | ・ | c. 35G>C | p. Arg12Pro |
| EPG5 | Hetero | ・ | ・ | ・ | C .6263dupT | p. Leu2088fs |
| STK4 | Hetero | ・ | ・ | ・ | c. 35+8G>A | ・ |
| C8A | Hetero | rs56334452 | 0.0003 | 0.000256 | c. 1654A>G | p. Arg552Gly |
| C5 | Hetero | rs772788429 | 8.24E-06 | 0.000012 | c. 2737C>T | p. Leu913Phe |

**Table S2**. List of the mutations in patients with IRAK4 deficiency

| Nucleotide | Protein | Position | Reference |
| --- | --- | --- | --- |
| 1-1096_40+23del | unknown | 5’UTR | (4, 20) |
| unknown | M1V | exon2 | (4, 23) |
| 34C>T | R12C | exon2 | (4, 19) |
| unknown | Y48* | exon2 | (4, 20) |
| 123_124insA | P42Tfs*3 | exon2 | (24) |
| 547C>T | R183* | exon5 | (4, 24) |
| 255_260dup6 | D86_87dup | exon3 | (22) |
| 573delA | M192Wfs*13 | exon5 | (25) |
| 593delG | G198Efs*7 | exon5 | (26) |
| 620_621delAC | T208Nfs*11 | exon5 | (4, 27) |
| 631delG | A211Qfs*1 | exon5 | (20) |
| 821delT | L274Pfs*13 | exon7 | (3, 4) |
| 831+5G>T | unknown | intron7 | (4, 19) |
| 877C>T | Q293* | exon8 | (3, 4, 27) |
| 893G>A | G298D | exon8 | (26) |
| 897_900delCAAT | N300Ffs*43 | exon9 | (4) |
| 942-1481_1125+547del | unknown | intron9 | (20) |
| 1146delT | G383Dfs*14 | exon10 | (22) |
| 1175G>T | unknown | splicing site of exon9-10 | (21) |
| 1188+520A>G | unknown | intron10 | (4, 28) |
| 1189-1G>T | unknown | intron10 | (4, 28) |
| 1204G>T | E402* | exon11 | (4, 29) |
| 1240insA | I414Nfs*1 | exon11 | (20) |
| unknown | Y430* | exon11 | (21) |


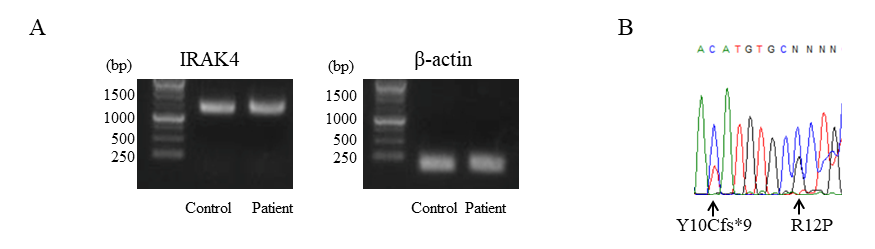


**Figure S1**

The reverse transcription PCR (RT-PCR)-based detection of *IRAK4* mRNA from PBMCs. (A) The expression of *IRAK4* mRNA in PBMCs from patient and unrelated healthy control. (B) Sanger sequencing of RT-PCR product from A to determine presence of both p.Y10Cfs*9 and p.R12P alleles at mRNA level.

**
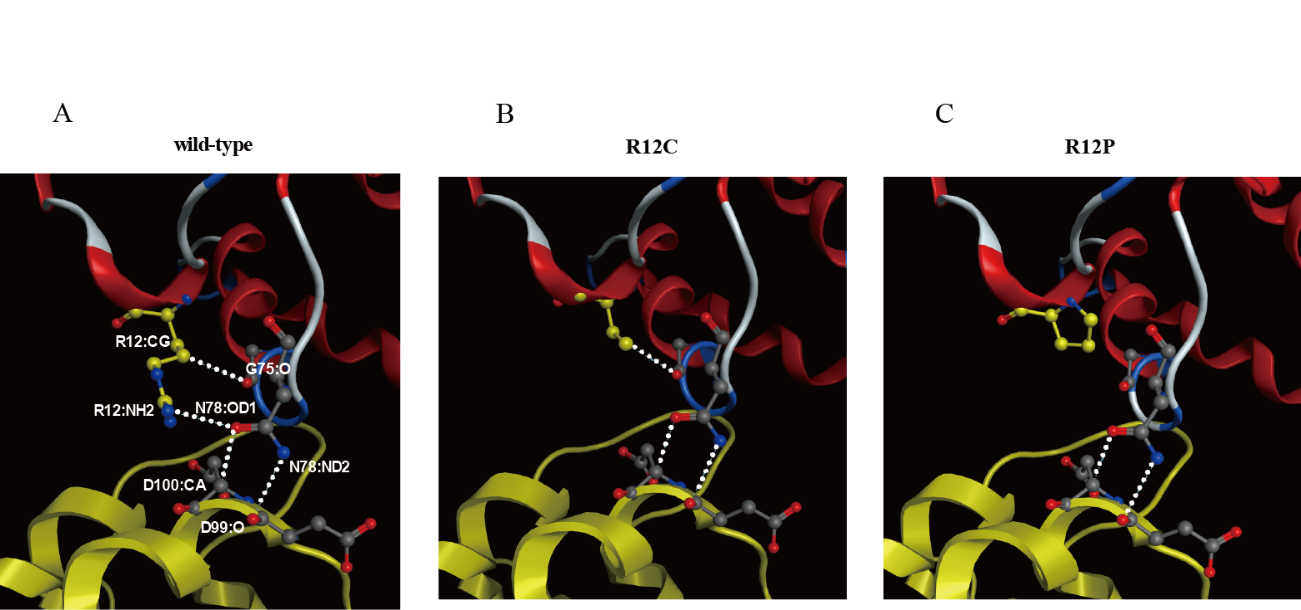
**

**Figure S2**

In *silico* analysis of the protein structure of IRAK4. 3D interaction models of IRAK4-death domain (DD) (red) with MyD88-DD (yellow). Residues of R12 appeared to be located on the surface of IRAK4-DD. A protein-protein interaction study was used to assess the mutational effect of these residues. (A) The recombinant proteins of IRAK4-DD+internal domain (ID) WT and MyD88-DD+ID WT formed a higher order oligomeric complex. The surface including R12 directly interacts with MyD88. (B) IRAK4 R12C could keep the molecular structure because of generating new inter molecular interaction with the subdomain of IRAK4, which was possibly formed among the side chains of IRAK4 R12C and G75. (C) IRAK4 R12P failed to interact with MyD88 due to loss of original inter molecular interaction of IRAK4 and lost the interaction to its subdomain.

**Supplemental materials and methods**

**Quantitative real-time-PCR and reverse transcriptional PCR (RT-PCR)**

Total RNA was extracted from PBMCs and was subjected to reverse transcription with random primers to generate cDNA. *IRAK4* mRNA levels were determined by quantitative PCR (qPCR) on the cDNA, with the CFX96 Touch Real-Time PCR Detection System (Bio Rad, USA). We used the following Taqman probes to determine the expression of *IRAK4* (Hs00211610_m1) and *GAPDH* (Hs99999905_m1) (Applied Biosystems, Waltham, Massachusetts, USA). The results were normalized with respect to the values obtained for the endogenous GAPDH cDNA. The RT-PCR was performed using primers spanning the entire coding region of the *IRAK4* gene. The condition of RT-PCR and primers used are available on request.

**Flow cytometry**

To investigate TNF-α production in response to lipopolysaccharide (LPS) stimulation, PBMCs were stimulated with 100 ng/ml of LPS (Invivogen) for 4 h. The cells were then stained with anti-CD14 antibodies and subjected to intracellular staining of TNF-α using a Fixation/Permeabilization Solution Kit with BD GolgiStop^TM^ (BD Becton, Dickinson and Company, Franklin Lakes, New Jersey, USA). The analysis gate was set for monocytes by forward and side scatter, and CD14 expression. Expression of intracellular TNF-α in monocytes was analyzed using flow cytometry (18). To assess IRAK4 protein expression, PBMCs were suspended at a density of 10^4^ cells/μl in RPMI supplemented with 10% FBS. They were then fixed and permeabilized according to the BD Phosflow protocol (Protocol Ⅲ) and stained with PE-conjugated anti-CD3, FITC-conjugated anti-CD4, CD8, CD19, CD14, Alexa 647-conjugated IRAK4 (BD Becton, Dickinson and Company), and anti-CD19 (BioLegend, San Diego, California, USA) antibodies. The stained cells were subjected to flow-cytometry analysis.

**Immunoblot analysis**

The HEK293T cells were maintained in DMEM supplemented with 10% FBS. The cells were harvested and plated at a density of 2.5×10^5^ cells/ml in six-well culture plates. After incubation for a further 24 h, plasmid DNA (5 μg/well) carrying the WT or a mutant *IRAK4* allele was introduced by lipofection using lipofectamine LTX (Thermo Fisher Scientific, Waltham, Massachusetts, USA). The transfected cells were incubated for 24 h, and then subjected to immunoblot analysis. Immunoblot analysis was performed as previously described (12). The following antibodies were used as the primary antibody; anti-FLAG M2 monoclonal antibody (Sigma-Aldrich, Saint Louis, MO, USA) and an anti-β-actin antibody (Sigma-Aldrich).

**Toll-like receptor (TLR) testing of patient fibroblasts**

To assess the impact of the *IRAK4* mutation on TLR signaling, we analyzed SV40 immortalized skin fibroblasts (SV40 fibroblast) from the patient as previously described (13). We also used SV40 fibroblasts from a patient with IRAK4 deficiency harboring a homozygous Q293* mutation as a disease control. Briefly, SV40 fibroblasts were stimulated with various agonists of TLRs. Twenty-four hours after stimulation, the supernatant was corrected and subjected to ELISA to detect IL-6 production. The experiments were performed in triplicate and two independent experiments were performed to confirm the results.
